# Muscle Fatigue Investigation Using sEMG Signals and a DAQ Card Data Acquisition System in LabVIEW

**DOI:** 10.3390/s26165051

**Published:** 2026-08-09

**Authors:** Zbigniew Krawiecki, Piotr Kuwałek

**Affiliations:** Institute of Electrical Engineering and Electronics, Poznan University of Technology, 61-138 Poznan, Poland

**Keywords:** surface electromyography (sEMG), muscle fatigue, LabVIEW, virtual instrumentation, data acquisition (DAQ), time-domain parameters, frequency-domain analysis, biosignal

## Abstract

The aim of this study is to implement a custom-designed data acquisition system with a DAQ card, based on the virtual instrument concept in the LabVIEW environment, to investigate and analyze muscle fatigue using surface electromyography (sEMG) signals. The experiment was conducted as a pilot case study on a healthy volunteer, where sEMG signals from the biceps brachii muscle were collected during cyclic weighted exercises. Signal registration was performed across three distinct states: no fatigue, moderate fatigue, and high fatigue. The developed measurement system enabled signal acquisition, filtering, and analysis through both online processing and post-processing. Time-domain parameters (ARV, RMS, $U_{\max}$) and frequency-domain parameters (ΣPS, MNF, MDF) were determined from three series of measurements. An analysis of parameter changes was conducted both within and between the series. The results indicated that with the onset of muscle fatigue, the participant exhibited a decrease in amplitude parameters and a shift in the power spectrum toward lower frequencies. Frequency-domain parameters, particularly MNF, exhibited higher diagnostic sensitivity than amplitude parameters. The obtained results confirm the technical feasibility of the developed virtual instrument for sEMG signal analysis. Furthermore, they suggest its potential utility for objective muscle condition assessment, establishing an engineering baseline for future research.

## 1. Introduction

This study constitutes a pilot research focused on the development and preliminary verification of a measurement setup for the acquisition and analysis of surface electromyography (sEMG) signals with online processing and post-processing capabilities. The results obtained provide a foundation for further system development and expansion of research to a larger subject population.

Surface electromyography is one of the most commonly utilized methods for assessing skeletal muscle activity. It is applied in biomechanical and rehabilitation studies, as well as in biomedical engineering [1,2,3,4]. This method enables the non-invasive recording of bioelectric signals generated by active muscle motor units [3,4,5]. Electrodes placed on the skin surface are used to acquire the signal. The recorded sEMG signals find wide application in the analysis of muscle activity during physical exercise and in the assessment of muscle fatigue [6,7,8]. Muscle fatigue is defined as a transient decrease in the muscle’s ability to generate force or maintain a specified torque value during prolonged physical activity [9]. Numerous studies have demonstrated that progressive muscle fatigue leads to characteristic changes in electromyographic signal [10,11,12]. These signal changes can be utilized as objective indicators of muscle fatigue. In the time-domain analysis of sEMG signals, parameters such as root mean square (RMS), maximum signal value ($U_{\max}$), and average absolute value (ARV) are applied, serving as measures of muscle activation intensity [13,14]. In contrast, in the frequency domain, the mean frequency (MNF) and median frequency (MDF) of the spectrum are utilized most frequently. These parameters exhibit a downward trend with increasing muscle fatigue [10,11].

In recent years, measurement systems have been advanced that enable the acquisition and simultaneous analysis of sEMG signals. These solutions integrate the hardware layer responsible for signal registration with its processing algorithms. LabVIEW (Laboratory Virtual Instrument Engineering Workbench) serves as an example of an environment for developing such systems. This platform enables the construction of measurement applications in the form of Virtual Instruments, integrating data acquisition, signal processing, and result visualization functions [7,15,16]. Additionally, tools such as the LabVIEW Biomedical Toolkit enable the implementation of advanced biomedical signal processing operations [17]. The literature describes numerous systems that utilize LabVIEW for EMG signal analysis. Hao et al. presented a system that enables the simultaneous recording of sEMG signals and the grip strength of the hand [18]. Shakya et al. proposed a solution for analyzing neck muscle fatigue using time- and frequency-domain parameters [19]. Gehlot et al. developed a muscle activity monitoring system integrated with IoT technology [20], while De la Pena et al. presented a wireless sEMG system for analyzing muscle fatigue during dynamic contractions [21].

Despite the growing number of solutions, relatively few studies have yet to present comprehensive measurement systems based on the concept of virtual instruments that enable simultaneous acquisition, processing, and analysis of sEMG signals using DAQ cards and LabVIEW.

The objective of this study was to design, test and evaluate a virtual instrument developed in the LabVIEW environment to investigate muscle fatigue based on sEMG signals, as part of a functional pilot testing in a single participant. The signal was acquired using a data acquisition system with a DAQ card during physical exercise. The main contribution of this work is the development of an integrated virtual measurement instrument in the LabVIEW environment for the analysis of surface electromyography (sEMG) signals and the control of the acquisition process with a DAQ card. The system enables simultaneous acquisition, signal processing and calculation of key parameters in the time and frequency-domains (ARV, RMS, $U_{\max}$, ΣPS, MNF, MDF) along with exploratory statistical analysis. To demonstrate the capabilities of the system, these parameters were utilized for the objective assessment of muscle fatigue during physical exercise in a single subject. The proposed solution constitutes a practical tool for monitoring muscle activity and highlights the potential of virtual measurement instrumentation. The presented results are intended to confirm the preliminary use of the proprietary virtual instrument developed in LabVIEW for the acquisition and analysis of sEMG signals regarding the investigation of muscle fatigue during dynamic contractions.

## 2. Materials and Methods

### 2.1. Experimental Procedure

The experiment was conducted on a healthy volunteer: 54 years old, without upper limb injuries, who is also the author of this work. Before measurement, the skin over the area of the biceps brachii muscle was washed with isopropyl alcohol to reduce the electrode–skin contact impedance [22,23]. The surface electrodes were placed along the course of the muscle fibers at a distance of 2 cm from each other, while the reference electrode was located outside of the investigated muscle, in accordance with the recommendations for sEMG measurements [3,24]. The study consisted of performing cyclic flexions and extensions of the forearm at the elbow joint with a 5 kg load in a seated position. The exercises were performed continuously at a constant movement pace. The signal recording was conducted in three series corresponding to different levels of muscle fatigue:

- series 1—no fatigue,
- series 2—moderate fatigue,
- series 3—high fatigue.

For each series, five measurement repetitions were performed. The recordings for the second and third series were initiated based on the subjective perception of the subject. Although the lack of an unequivocal objective fatigue protocol (e.g., torque monitoring, MVC normalization, or verification using the standardized Borg CR-10 scale) constitutes a methodological limitation, the primary objective of this pilot implementation was to test the technical capabilities of the virtual instrument in tracking signal alterations [23].

In subsequent sections, the results obtained in LabVIEW are presented for each series, and the calculated values of the monitored parameters are summarized in tables. The results obtained were used to assess the perceived level of muscle fatigue.

### 2.2. Measurement Setup with sEMG Data Acquisition Software

To acquire the sEMG signal, a measurement setup was applied, consisting of:

- an amplifier and bandpass filter module,
- a myDAQ data acquisition card (National Instruments),
- a computer with software developed in the LabVIEW environment.

A two-stage amplifier adjusted the voltage level of the sEMG signal to the input range of the acquisition card. The first stage was implemented using an INA128 instrumentation amplifier (Texas Instruments, Dallas, TX, USA), while the second stage utilized an OPA2134 operational amplifier (Texas Instruments, Dallas, TX, USA). The total gain of the system was $K_{U}$ = 700 *v*/*v* and was determined experimentally.

**Figure 1:**
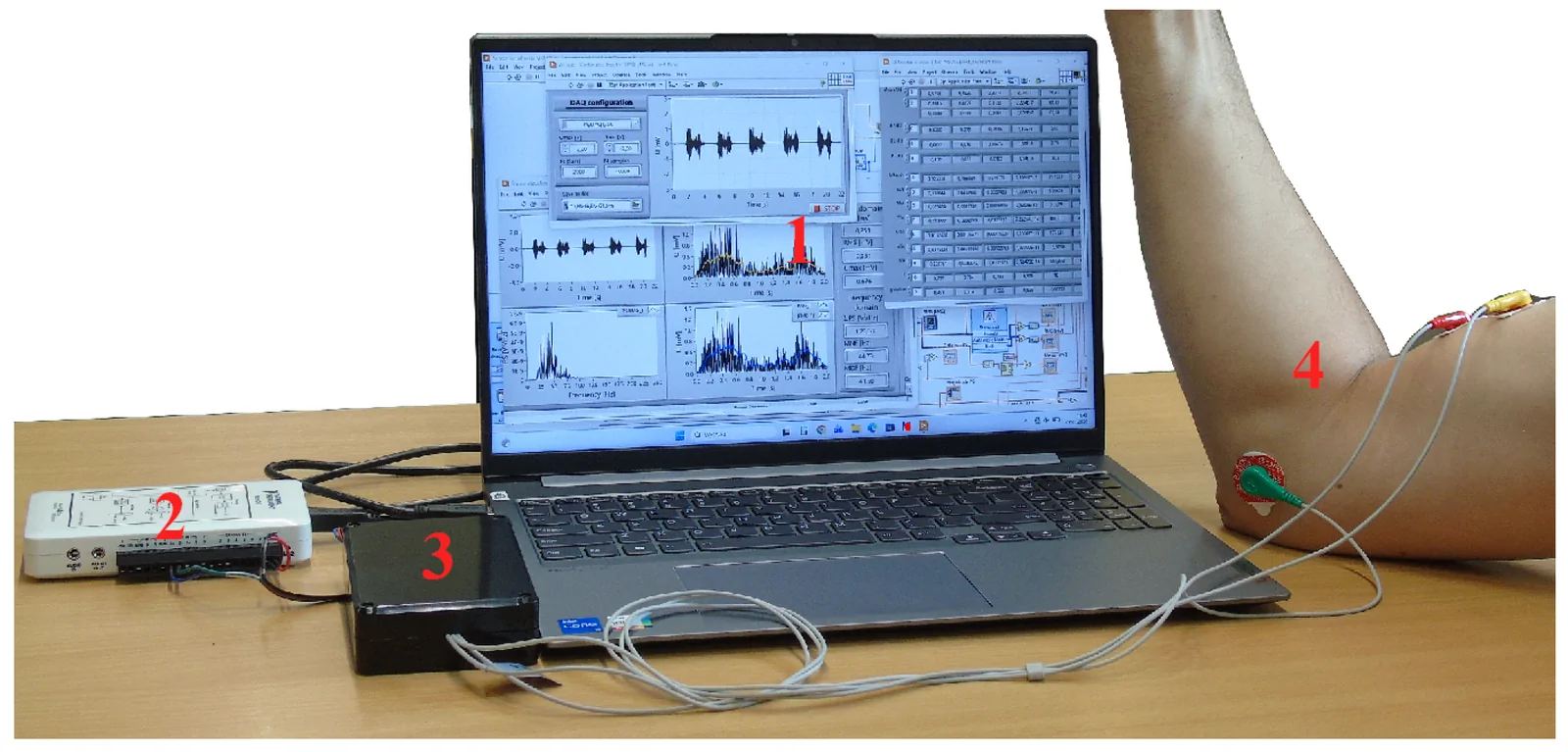
Developed measurement setup for sEMG signal recording, where: 1—computer with LabVIEW software (version 2025Q3, National Instruments, Austin, TX, USA), 2—NI myDAQ data acquisition card, 3—amplifier and bandpass filter module, 4—disposable conductive electrodes placed on the subject’s skin.

The applied bandpass filter in the Sallen–Key topology (40 dB/decade slope) limited the signal bandwidth to the range of 15–500 Hz, which complies with the typical frequency range of sEMG signals [6,25]. Filtration allowed for reduction of the DC component and motion artifacts. The frequency response of the system was verified experimentally. The amplifier and filter module were tested in a laboratory at Poznan University of Technology using a DG1022Z generator and a DS1054Z oscilloscope (both from Rigol Technologies, Suzhou, China). The amplifier-filter module was powered from the +5 V output of the myDAQ card (National Instruments, Austin, TX, USA). This output is intended to power external circuits and sensors. Disposable electrodes with conductive gel were connected to the input of the module. After amplification and filtration, the analog signal was acquired using the myDAQ card equipped with a 16-bit A/D converter (ADS8319, Texas Instruments, Dallas, TX, USA). The developed measurement setup is presented in Figure 1.

The recording was conducted in a differential configuration with a sampling rate of 2000 Sa/s, which satisfies the sampling requirements for sEMG signals [25]. A total of 40,000 samples were recorded during the measurement. Within the LabVIEW environment, a virtual measurement channel was developed to control acquisition, including visualization, processing, and signal storage.

**Figure 2:**
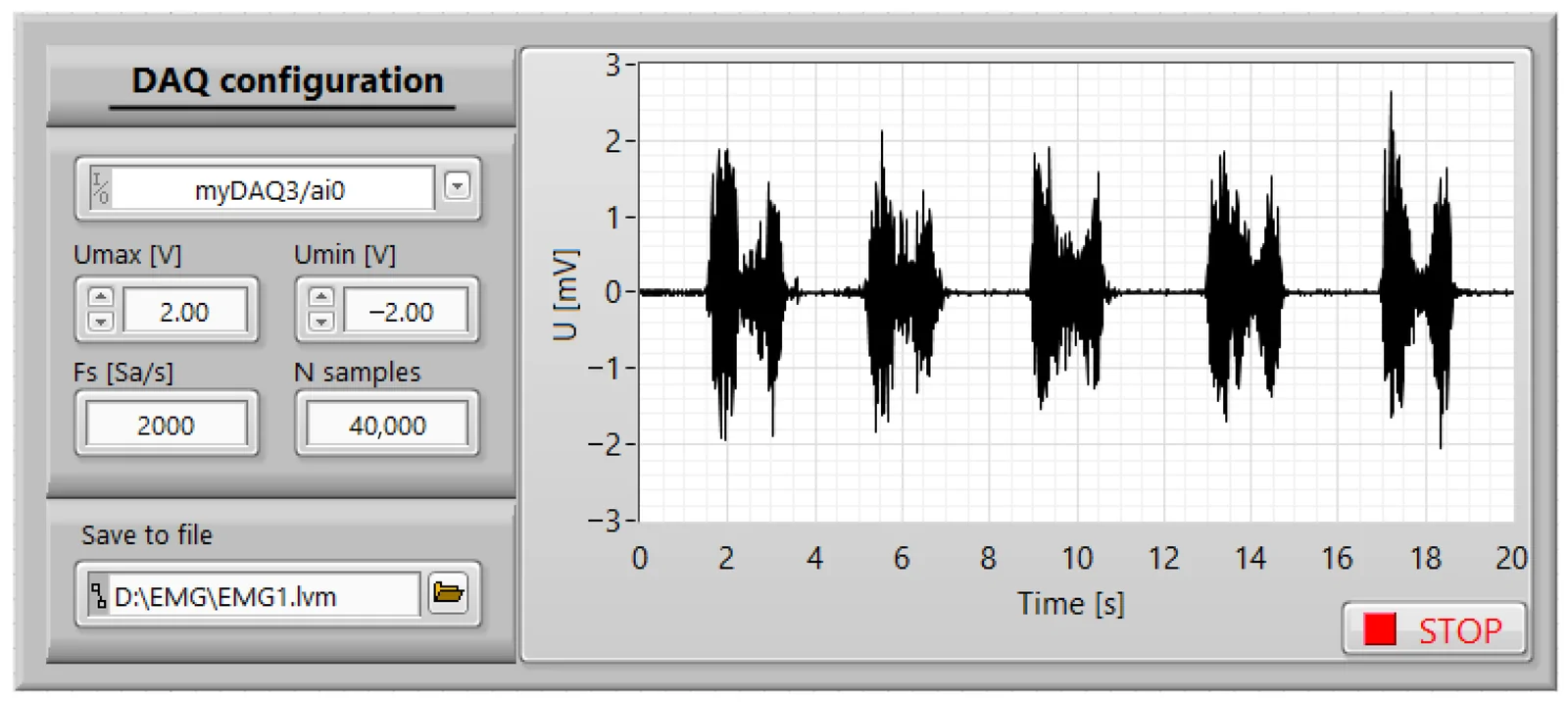
LabVIEW application panel for sEMG signal acquisition.

The program panel for controlling the DAQ card, along with the visualization of the recorded signal, is presented in Figure 2.

The panel enables configuring the measured voltage range, sampling rate, and number of samples, as well as visualizing the recorded signal. Subsequently, the signal is processed to determine parameters in the time and frequency-domain.

**Figure 3:**
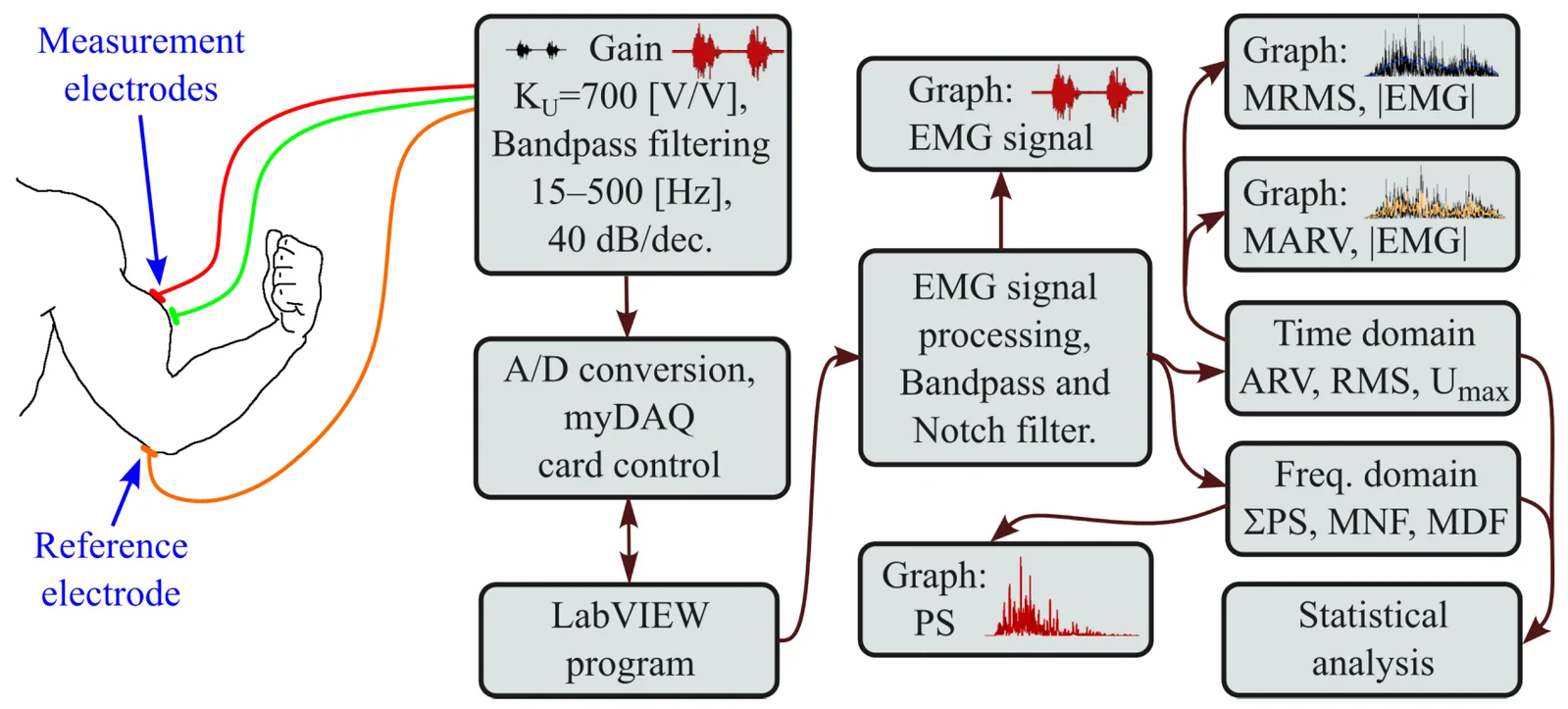
Block diagram of EMG signal processing in LabVIEW.

The LabVIEW program was developed in the form of panels assigned with specific tasks: signal acquisition, processing, parameter determination, statistical calculations, and result visualization. A general schematic of the signal processing workflow is presented in Figure 3.

The block diagram includes the following stages of signal processing: acquisition, filtering, envelope smoothing (Moving RMS—MRMS, Moving ARV—MARV), determination of time-domain parameters (ARV, RMS, $U_{\max}$) and frequency-domain parameters (ΣPS, MNF, MDF), as well as result visualization. In the algorithm, software digital filtration was applied using a Butterworth filter (15–500 Hz, 40 dB/decade) to reduce motion artifacts and DC component. Additionally, a Notch filter (50 Hz, −3 dB bandwidth of 0.2 Hz) was implemented to eliminate power-line interference [25].

For the analysis, sEMG signals with a duration of 1.8 s were extracted. In the spectral analysis, a Hanning window was utilized, which reduces the effect of spectral leakage and improves the precision of estimating the frequency parameters [26,27]. In the time and frequency domains, parameters were determined and compared within and between series (statistical analysis).

**Figure 4:**
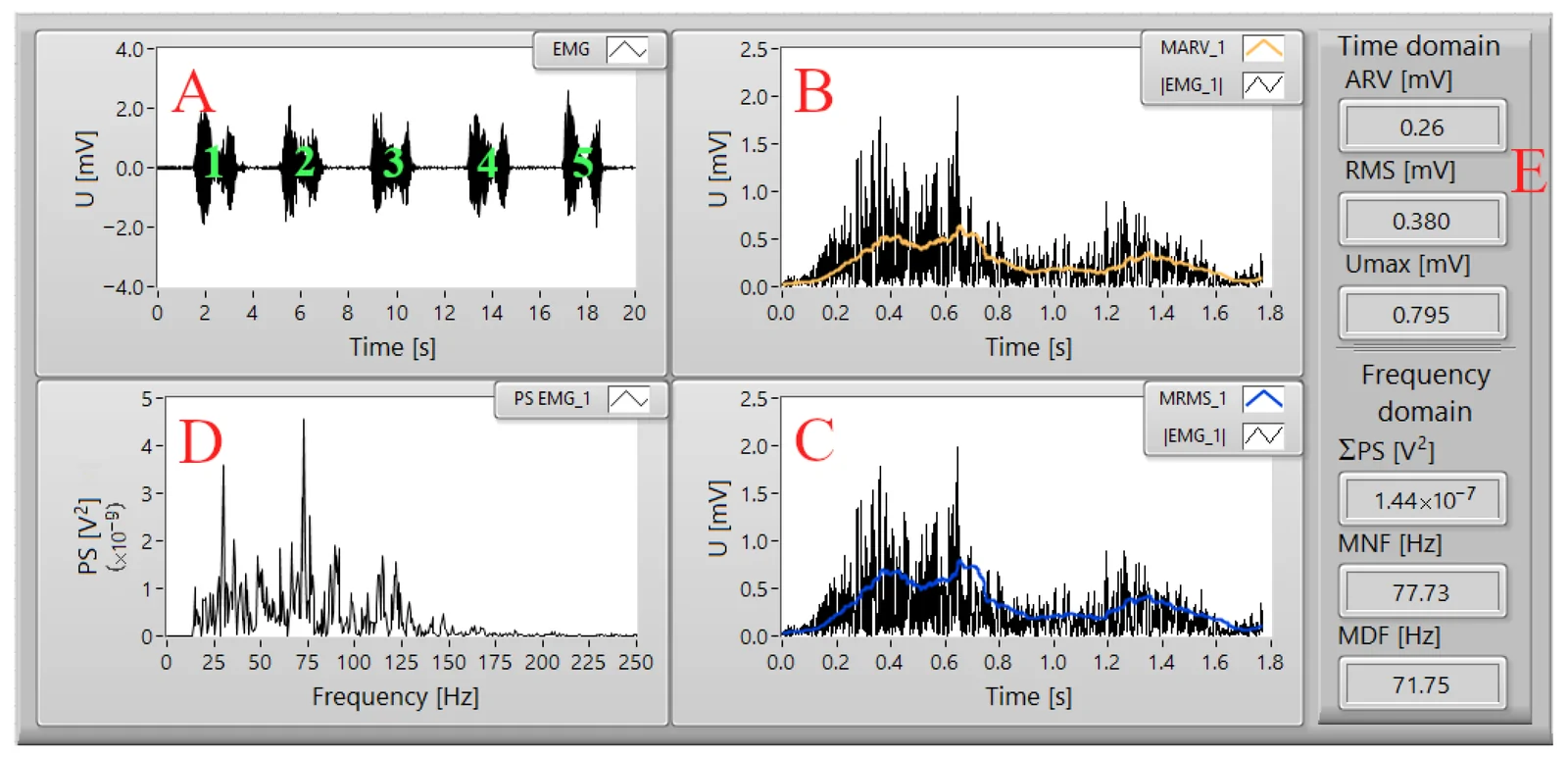
LabVIEW program panel with signal processing results: (**A**) total signal recorded during a series of five cycles of lifting and lowering the load; (**B**) absolute value of the signal envelope |EMG_1| and the envelope MARV_1 for the first cycle of this series; (**C**) again |EMG_1| and the envelope MRMS_1 for the first cycle of this series; (**D**) power spectrum of the signal for the first cycle, showing the energy concentration in the 20–160 Hz range; (**E**) determined time- and frequency-domain parameters for the first cycle.

An exemplary LabVIEW program panel featuring selected signal processing components is presented in Figure 4.

In the next stage, the results for the second, third, fourth, and fifth cycles of lifting and lowering the load are presented on displays B, C, D and E in Figure 4.

### 2.3. Signal Processing Methods

The parameters were determined for signal segments with a duration of 1.8 s, corresponding to a full movement cycle (flexion–extension). In the time domain, the following were determined within LabVIEW:

- the absolute value of the signal |EMG|,
- the signal envelope using the MARV moving average [Equation (1)],
- the average absolute value (ARV) for the concentric and eccentric phases [Equation (1)],
- the signal envelope using the MRMS moving RMS [Equation (2)],
- the root mean square (RMS) value for the concentric and eccentric phases [Equation (2)],
- the maximum signal amplitude ($U_{\max}$) for the smoothed moving RMS envelope.

These parameters are commonly applied as measures of muscle activation and fatigue levels [13,14,28]. The moving average of the rectified signal (MARV) and the average absolute value (ARV) were determined using Equation (1), considering the number of samples *N*:

(1) $$\mathrm{MARV}=\frac{1}{N}\sum_{i=1}^{N}\left|x_{i}\right|,$$

where $x_{i}$ represents the sEMG signal samples. In contrast, the RMS and MRMS were determined using Equation (2):

(2) $$\mathrm{RMS}=\sqrt{\frac{1}{N}\sum_{i=1}^{N}x_{i}^{2}},$$

where *N* is the number of samples in the time window.

In the frequency domain, the following were determined within LabVIEW:

- the total signal power (ΣPS),
- the mean frequency (MNF),
- the median frequency (MDF).

The mean frequency (MNF) of the signal spectrum was determined using Equation (3):

(3) $$\mathrm{MNF}=\frac{\sum_{j=1}^{M}f_{j}P_{j}}{\sum_{j=1}^{M}P_{j}},$$

where $f_{j}$ denotes frequencies, $P_{j}$ the corresponding values of the power spectrum, and M is the number of spectral points.

The median frequency (MDF) is defined as the frequency that divides the total power spectrum into two equal halves, as expressed in Equation (4):

(4) $$\mathrm{MDF}=f\left(k\right),k:\sum_{j=1}^{k}P\left(f_{j}\right)=\frac{1}{2}\sum_{j=1}^{M}P\left(f_{j}\right),$$

where *k* denotes the frequency index at which half of the total signal spectral power is reached.

The MNF and MDF parameters are widely used as indicators of muscle fatigue and tend to decrease with increasing fatigue [10,11]. Due to the pilot nature of the study based on a case report (n=1), the statistical analysis was treated exclusively as exploratory. It was performed within the LabVIEW environment using a one-way repeated measures analysis of variance (ANOVA) for technical replicates, as well as a paired *t*-test. For all tests, the significance level was set at α=0.05 [7,23]. Statistical analysis was applied to assess the variability of the intra- and inter-series signal for the investigated subject.

## 3. Results

### 3.1. Time- and Frequency-Domain Signal Parameters

The signals recorded from the biceps brachii muscle and subsequently analyzed corresponded to three states: no fatigue (series 1), moderate fatigue (series 2) and high fatigue (series 3). The signals were acquired during dynamic exercises. Changes in amplitude parameters (ARV, RMS, $U_{\max}$) and frequency parameters (MNF, MDF), as well as total signal power (ΣPS), were evaluated.

**Figure 5:**
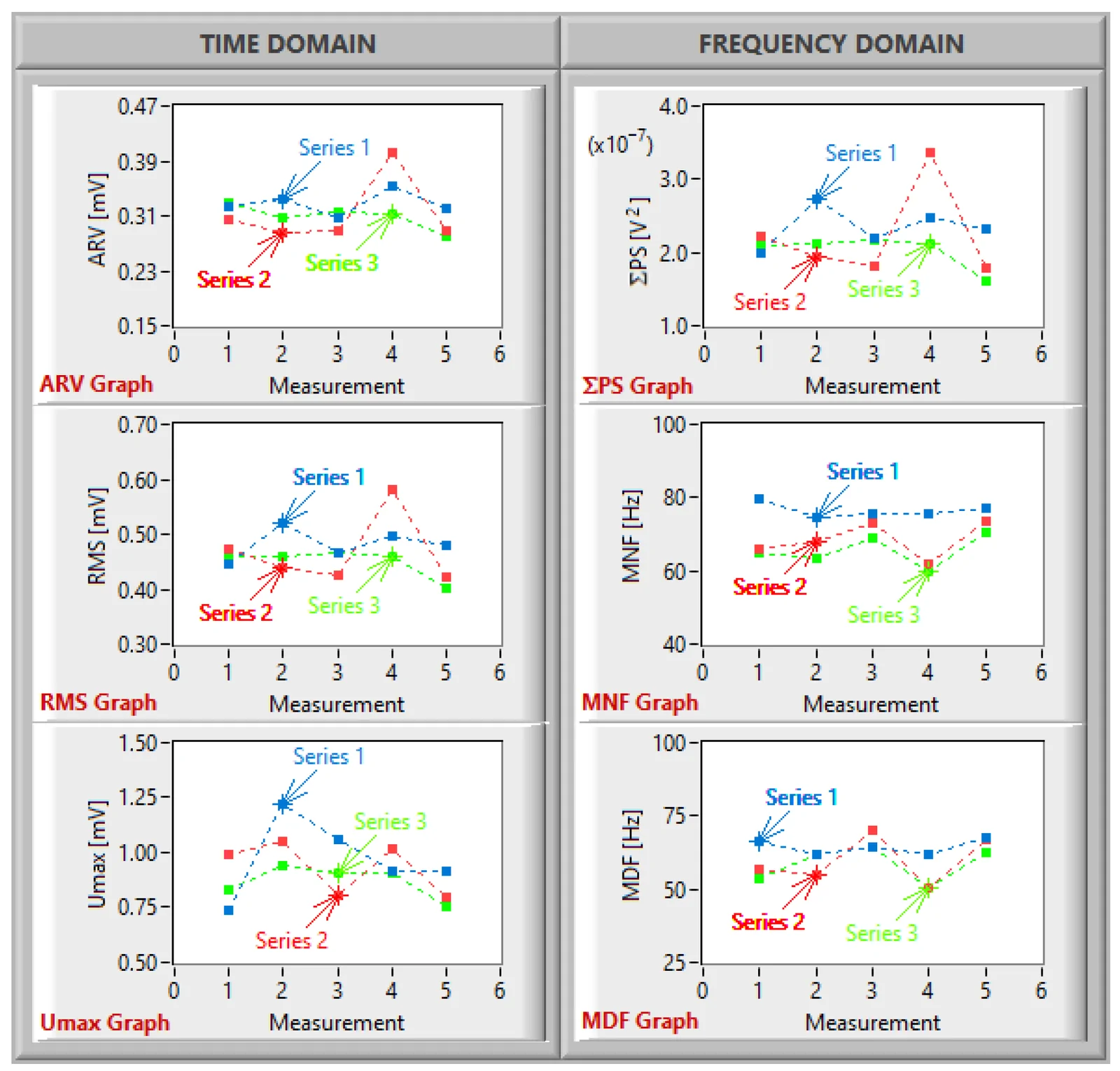
Panel with the results of the determined time- and frequency-domain parameters for series 1, 2, and 3.

The parameters calculated by the software are presented on the LabVIEW plotting panel, as shown in Figure 5.

**Figure 6:**
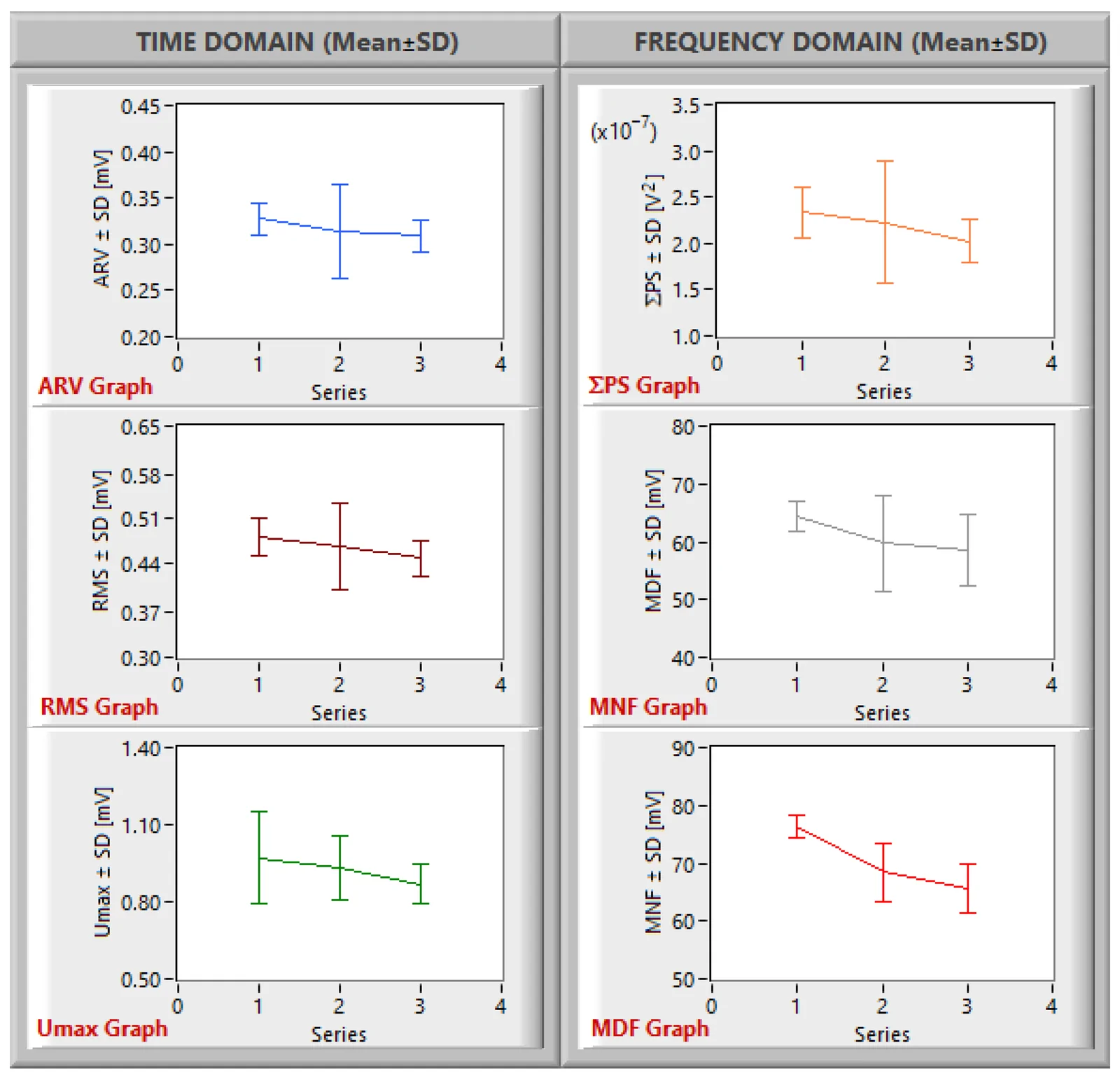
Panel with the visualization of the mean value and standard deviation for parameters from series 1, 2, and 3.

For each parameter determined (ARV, RMS, $U_{\max}$, ΣPS, MNF, MDF), the mean value of the series and the standard deviation (SD) were calculated and presented graphically in the LabVIEW panel (Figure 6).

The sEMG signal waveform indicates significant changes in amplitude and a shift of the power spectrum toward lower frequencies. The decrease in MNF and MDF, the reduction in ΣPS, and the characteristic changes in $U_{\max}$, ARV, and RMS observed in subsequent series confirm the functionality of the system to effectively record trends related to the progression of muscle fatigue during dynamic contractions in the studied participant.

### 3.2. Analysis of Parameter Changes Within Series

An evaluation of changes in the parameters of the sEMG signal is conducted both within and between series.

#### 3.2.1. Analysis of Changes Within Series 1 (Beginning vs. End)

For the results summarized in Table 1, minor changes in values are noticeable, which, in the case of this test, may reflect an adaptive trend of the muscle studied to dynamic physical exercise. A significant increase in signal amplitude $U_{\max}$ (24%) was observed after envelope smoothing using MRMS.

#### 3.2.2. Analysis of Changes Within Series 2 (Beginning vs. End)

Within the results obtained for series 2 (Table 2), a noticeable decrease is observed in the values of the parameters of the time-domain, while there is an increase in MNF and MDF in the frequency domain. This may suggest local changes in the recruitment of faster motor units in the studied participant. A significant in-stability of the signal is also noticeable.

#### 3.2.3. Analysis of Changes Within Series 3 (Beginning vs. End)

The values determined for series 3 (Table 3) indicate a clear decrease in all amplitude parameters, accompanied by an increase in MNF and MDF.

The greatest variability of time-domain parameters was recorded for the $U_{\max}$ amplitude in series 1 (±0.18 mV), whereas for ARV (±0.05 mV) and RMS (±0.07 mV) in series 2. Regarding the parameters of the frequency-domain, the highest variability was obtained in series 2: ΣPS (±0.7 × $10^{-7}$ $V^{2}$), MNF (±4.9 Hz) and MDF (±8.3 Hz), indicating instability of both the recorded signal and muscle activity during the moderate fatigue phase for this test trial. Within series 2 and 3, an increase in MNF and MDF values was observed, which might appear contradictory to the classical model of muscle fatigue. However, in this specific case study, this phenomenon can be linked to the non-stationary nature of the sEMG signal during dynamic contractions, as well as to the individual strategy of motor unit recruitment and derecruitment. The literature emphasizes that during dynamic contractions, spectral parameters can exhibit significant variability resulting from changes in muscle geometry, conduction velocity, and motor unit recruitment strategies [4,29,30]. Furthermore, the dependence of MNF and MDF parameters on fatigue is non-linear and can be modulated by the level of force generated [10,31].

### 3.3. Analysis of Parameter Changes Between Series

sEMG signal analysis revealed systematic changes in both time and frequency-domain parameters with increasing muscle fatigue (Table 4). Within the framework of the described case study, these changes are monotonic and consistent with the well-known physiological mechanisms of muscle fatigue. The amplitude parameters (ARV, RMS, $U_{\max}$) exhibited a gradual decrease, reflecting the trend of decreasing force-generating capacity observed in the studied participant, which is widely described in the literature [6,24].

The greatest change in the mean value was obtained within the time domain for $U_{\max}$. From the onset of physical exercise, this value decreased by approximately 11%. A similar trend was observed for RMS, with a decrease of approximately 6.8%, and ARV, with a decrease of approximately 5.6%. Variation analysis revealed relatively small standard deviations for ARV (0.02–0.05 mV) and RMS (0.03–0.07 mV), indicating good repeatability of the measurement. The exploratory statistical analysis (ANOVA) revealed no distinct intra-test differences between the series for the time-domain parameters ($U_{\max}$: F(2,8), *p* = 0.69; RMS: F(2,8), *p* = 0.47; ARV: F(2,8), *p* = 0.56).

The frequency-domain parameters exhibited a distinct downward trend: ΣPS values decreased by approximately 13% between series 1 and 3, MNF by approximately 14%, and MDF by approximately 9%. For ΣPS, no distinct intra-test differences were found between fatigue levels (*p* > 0.05). The MNF and MDF parameters demonstrated a shift in the power spectrum to lower frequencies, which is a well-documented muscle fatigue effect resulting from a decrease in the conduction velocity of muscle fibers [9,10,11,32]. The exploratory statistical analysis (ANOVA) revealed distinct inter-series variability for MNF (F(2,8) = 13.7, *p* = 0.0027). For MNF, further exploratory pairwise comparisons (series 1 vs. 2, series 2 vs. 3, and series 1 vs. 3) are conducted using a paired *t*-test. Distinct intra-test differences are obtained between series 1 and 2 (*p* = 0.028), series 2 and 3 (*p* = 0.010), and series 1 and 3 (*p* = 0.005). For MDF, the significance threshold was not reached (F(2,8) = 3.65), *p* = 0.075); however, the observed downward trend indicates its potential diagnostic value. All statistical indicators presented (including *p*–values) should be interpreted exclusively as a measure of the system’s measurement stability and reproducibility for the investigated subject, rather than as population-based conclusions.

## 4. Discussion

The results obtained indicate a clear influence of muscle fatigue on the characteristics of the electromyographic signal. The observed changes affected both both amplitude and frequency parameters, although their diagnostic sensitivity varied.

The time-domain parameters (ARV, RMS, $U_{\max}$) exhibited a general downward trend with increasing muscle fatigue, which may reflect a reduction in muscle power capacity and alterations in motor unit activation [6,24]. However, the lack of statistical significance for these parameters points to their limited sensitivity, particularly during the initial stages of fatigue. This phenomenon is consistent with reports from the literature that state that changes in the amplitude of the signal in sEMG can be ambiguous and highly dependent on the type of exercise (static vs. dynamic) and the recruitment strategies of the motor unit [13,14,33,34].

Significantly more unambiguous changes were observed in the frequency domain. The decrease in the mean frequency (MNF) of the spectrum and the downward trend of the median frequency (MDF) indicate a power spectrum shift toward lower frequencies. This effect is well-documented and is mainly the result of a decrease in muscle fiber conduction velocity as well as metabolic changes that occur within the muscle [9,10,11,33,35]. A particularly notable finding is the high sensitivity of the MNF parameter, for which significant differences were demonstrated between the non-fatigued and fatigued states. This confirms reports indicating that frequency-based indices are a more reliable tool for assessing muscle fatigue than amplitude-based parameters, particularly during cyclic muscle contractions [7,10,34]. Although the MDF parameter did not reach statistical significance, the observed trend suggests its potential diagnostic utility. Increased variability in spectral parameters (MNF, MDF) during dynamic contractions is a widely documented phenomenon [29,31,34,36,37]. This contrasts sharply with static isometric tests. During cyclic flexion and extension movements of the forearm, the muscle geometry changes continuously [37]. This shifting configuration modifies the distance between the motor units and the electrodes, directly affecting the frequency components of the sEMG signal [35,37]. As fatigue progresses during dynamic exercise, the central neuromuscular system modifies recruitment strategies, periodically activating fast-twitch units to maintain the constant torque required to lift the 5 kg load [38]. This adaptive recruitment introduces instantaneous high-frequency components into the signal, explaining local fluctuations and transient increases in the MNF and MDF values recorded in series corresponding to moderate and high fatigue [14]. The non-stationarity of dynamic sEMG signals complicates the linear interpretation of traditional Fourier spectral indices [37]. The observed intra-test variability is most likely the result of an interplay between two mechanisms: first, a decrease in muscle fiber conduction velocity induced by metabolic changes [33,34], and second, adaptive alterations in motor unit recruitment strategies by the nervous system [38]. The combined impact of these processes provides a reason for the variability of spectral parameters recorded during dynamic trials. The differences between the first and last repetitions within the same series indicate the dynamic nature of this process which is consistent with previous research on fatigue during dynamic exercise [6,14,37]. The increased variability of the parameters, particularly in the series corresponding to moderate fatigue, indicates the presence of an adaptive phase. In this phase, the neuromuscular system compensates for developing fatigue by modifying motor unit recruitment and their discharge frequencies [4,23,30,38,39].

The results obtained confirm that muscle fatigue develops progressively and can be observed even within short movement sequences. Signal analysis should encompass both the time and frequency domains, with particular emphasis on spectral analysis as a more sensitive method to evaluate muscle fatigue [7,26].

A significant limitation of the conducted study is the small sample size (n=1), and the acquired measurements constitute technical replicas. Consequently, the obtained *p*-values and the results of the ANOVA and *t*-tests must be interpreted with great caution. They do not provide evidence of statistical significance in a biological or population-based sense; rather, they merely describe the intra-test variability and measurement reproducibility of the developed system for this specific participant. For this reason, the statistical analysis was utilized exclusively for exploratory and engineering purposes. Confirming these trends requires further studies on a larger group of participants.

Another significant limitation of this pilot study is that the classification of fatigue levels (none, moderate, and high) was based exclusively on the subjective assessment of the participant rather than a standardized fatigue protocol. Without objective metrics—such as real-time force or torque measurements, maximum voluntary contraction (MVC) normalization, or structured Borg scale assessments—the exact thresholds between the three series remain uncalibrated and susceptible to intra-individual variability [40]. Consequently, the recorded series represent qualitative fatigue transitions rather than precisely quantified physiological thresholds. This baseline approach was adopted due to the exploratory nature of the study, which prioritized technical verification of the LabVIEW-based instrumentation setup. The developed proprietary setup configured as a virtual measurement instrument with a DAQ card and LabVIEW software can be utilized to implement an objective muscle fatigue control method. The hardware and software expansion of the setup with an additional channel for torque measurement is feasible and will be undertaken in future research.

## 5. Conclusions

The main achievement of this work was the design, implementation, and technical verification of an integrated virtual measurement device in the LabVIEW environment, working with a DAQ card for sEMG signal recording and processing. The developed software efficiently implements the data acquisition process and incorporates digital processing algorithms enabling automatic determination of key sEMG signal parameters in the time and frequency domains.

In the pilot case study, the amplitude parameters ($U_{\max}$, ARV, RMS) showed a decreasing trend during dynamic contractions, but their diagnostic sensitivity proved limited, particularly in the initial stages of fatigue. Frequency indices (MNF, MDF) were shown to be more sensitive measures of changes that occur in the sEMG signal. In particular, the mean frequency spectrum (MNF) already showed significant intra-test shifts at the moderate fatigue stage. As fatigue progressed, a shift in the sEMG signal power spectrum towards lower frequencies and a decrease in total ΣPS signal power were observed. However, these changes reflect only the signal characteristics of the participant tested under specific dynamic exercise conditions and must be interpreted with caution.

It should be emphasized that the statistical metrics and *p*-values obtained from technical replicates are purely exploratory, confirming the internal consistency and repeatability of the developed virtual device, not population-based relationships. All results presented in this paper were automatically determined using the LabVIEW software developed.

Significant limitations include the single-subject sample size (n=1, one of the authors) and the classification of fatigue states based solely on the subjective assessment of the participant. The pilot nature of the study and the lack of objective methods for assessing fatigue, such as force or torque measurement, normalization to maximum voluntary contraction (MVC), or the use of the Borg scale, mean that the developed system should be considered a pilot solution and a basis for further development.

In future studies, it is recommended to expand the research group and expand the virtual measurement station with an additional channel for force or torque recording. It is also planned to develop software algorithms to analyze signal parameters as a function of the time window length, as well as to use advanced methods of non-stationary signal processing, such as the short-time Fourier transform (STFT) and wavelet analysis (Wavelet Transform).

## Figures and Tables

**Table 1:** Comparison of parameters within series 1 for the first and last recordings.

Parameter	First Recording	Last Recording	Relative Change
ARV [mV]	0.323	0.321	↓ (0.5%)
RMS [mV]	0.447	0.480	↑ (7.4%)
$U_{\max}$ [mV]	0.741	0.917	↑ (24%)
ΣPS [$V^{2}$]	2.00 × $10^{-7}$	2.31 × $10^{-7}$	↑ (15%)
MNF [Hz]	79.56	77.05	↓ (3.2%)
MDF [Hz]	66.10	67.80	↑ (2.6%)

**Table 2:** Comparison of parameters within series 2 for the first and last recordings.

Parameter	First Recording	Last Recording	Relative Change
ARV [mV]	0.305	0.289	↓ (5.3%)
RMS [mV]	0.473	0.421	↓ (11%)
$U_{\max}$ [mV]	0.992	0.797	↓ (20%)
ΣPS [$V^{2}$]	2.23 × $10^{-7}$	1.78 × $10^{-7}$	↓ (20%)
MNF [Hz]	65.98	73.48	↑ (11%)
MDF [Hz]	57.06	66.67	↑ (17%)

**Table 3:** Comparison of parameters within series 3 for the first and last recordings.

Parameter	First Recording	Last Recording	Relative Change
ARV [mV]	0.328	0.281	↓ (15%)
RMS [mV]	0.459	0.403	↓ (12%)
$U_{\max}$ [mV]	0.835	0.752	↓ (10%)
ΣPS [$V^{2}$]	2.10 × $10^{-7}$	1.62 × $10^{-7}$	↓ (23%)
MNF [Hz]	64.95	70.72	↑ (8.9%)
MDF [Hz]	53.67	62.71	↑ (17%)

**Table 4:** Comparison of mean parameter values between series.

Mean	Series 1	Series 2	Series 3	Change (1–2)	Change (2–3)	Change (1–3)
ARV [mV]	0.327	0.314	0.309	↓ (4.1%)	↓ (1.5%)	↓ (5.6%)
RMS [mV]	0.483	0.468	0.450	↓ (3.1%)	↓ (3.8%)	↓ (6.8%)
$U_{\max}$ [mV]	0.970	0.932	0.868	↓ (4.0%)	↓ (6.8%)	↓ (11%)
ΣPS [$V^{2}$]	2.34 × $10^{-7}$	2.22 × $10^{-7}$	2.03 × $10^{-7}$	↓ (4.9%)	↓ (8.7%)	↓ (13%)
MNF [Hz]	76.45	68.43	65.63	↓ (11%)	↓ (4.1%)	↓ (14%)
MDF [Hz]	64.41	59.77	58.53	↓ (7.2%)	↓ (2.1%)	↓ (9.1%)

## Data Availability

The data presented in this study are available on request from the corresponding author.
